# Author Correction: Seed dispersal by Martu peoples promotes the distribution of native plants in arid Australia

**DOI:** 10.1038/s41467-024-52253-1

**Published:** 2024-09-11

**Authors:** Rebecca Bliege Bird, Douglas W. Bird, Christopher T. Martine, Chloe McGuire, Leanne Greenwood, Desmond Taylor, Tanisha M. Williams, Peter M. Veth

**Affiliations:** 1 The Pennsylvania State University, Department of Anthropology, University Park, PA 16801 USA; 2 Department of Biology, Bucknell University, 1 Dent Drive, Lewisburg, PA 17837 USA; 3 Far Western Anthropological Research Group, 2727 Del Rio Pl, Davis, CA 95618 USA; 4 Dja Dja Wurrung Clans Aboriginal Corporation, Bendigo, Australia; 5 Martu Elder, Kulyakartu Aboriginal Corporation, 76 Wittenoom St, East Perth, WA 6004 Australia; 6 Department of Plant Biology, University of Georgia, 2502 Miller Plant Sciences, Athens, GA 30602-7271 USA; 7 The University of Western Australia, School of Social Sciences and ARC Centre of Excellence for Indigenous and Environmental Histories and Futures, Crawley, WA Australia

**Keywords:** Biodiversity, Archaeology, Biological anthropology, Social anthropology, Agroecology

**Correction to:** *Nature Communications* 10.1038/s41467-024-50300-5, published online 17 July 2024

The original version of this Article contained errors in Table 4 and its legend. In Table 4, cells labels were omitted for the cases in which: (i) the predictor was significant in the global model but not in the best model, and (ii) support for anthropogenic dispersal or anthropogenic engineering on presence or abundance. In the legend of Table 4, the significance of the grey shading was incorrectly described. The correct version of Table 4 is:

**Table:** 

	*Diversiflorum*		*Centrale*		*Eragrostis*		*Scaevola*	
	Presence	Abundance	Presence	Abundance	Presence	Abundance	Presence	Abundance
**Dispersal proxies**								
Site distance	Strong negative**	Negative**	Weak negative under high land use**	No effect	Negative under high land use**	Negative near ephemeral water**	Weak positive	Weak positive
Land use	Strong positive**	No effect	Weakly Positive under high use**	No effect	Weak Negative	No effect	No effect	No effect
Water permanence	Strong positive**	Strong positive**	No effect	No effect	No effect*	Negative close to sites**	No effect	No effect
Site type Major	Negative**	No effect	No effect	No effect	No effect*	No effect**	Negative	Positive
**Engineering proxies**								
Season Winter	No effect*	No effect	No effect	Negative	Negative	Positive**	No effect	No effect
Fire frequency	No effect	No effect	Strong positive*	Weak negative	No effect	No effect	No effect	No effect
TSF diversity	No effect*	No effect	No effect	No effect	No effect*	No effect	No effect	No effect

The correct version of the Table 4 legend is:

Table 4. Hypothesis test outcomes from the best model predicting presence and abundance. No effect = coefficient not in best model: dropping coefficient reduced AIC value of the model during model selection and ranking. Positive effect = covariate increased presence or abundance. Negative effect = covariate reduced presence or abundance. *: the predictor was significant in the global model but not in the best model. **: support for anthropogenic dispersal or anthropogenic engineering on presence or abundance.

This has been corrected in both the PDF and HTML versions of the Article.

In addition, several affiliation details were incorrect. Affiliation 1 for Rebecca Bliege Bird and Douglas W. Bird was incorrectly given as ‘Department of Anthropology, The Pennsylvania State University, University Park, PA, USA’ but should have been ‘The Pennsylvania State University, Department of Anthropology, University Park, PA 16801’. Affiliation 2 for Christopher T. Martine was incorrectly given as ‘Far Western Anthropological Research Group, Davis, CA, USA’ but should have been ‘Department of Biology, Bucknell University, 1 Dent Drive, Lewisburg, PA 17837’. Affiliation 3 for Chloe McGuire was incorrectly given as ‘Dja DjaWurrung Clans Aboriginal Corporation, Bendigo, VIC, Australia’ but should have been ‘Far Western Anthropological Research Group, 2727 Del Rio Pl, Davis, CA 95618’. Affiliation 4 for Leanne Greenwood was incorrectly given as ‘Kulyakartu Aboriginal Corporation, East Perth, WA, Australia’ but should have been ‘Dja Dja Wurrung Clans Aboriginal Corporation, Bendigo, Australia’. Affiliation 5 for Desmond Taylor was incorrectly given as ‘Department of Plant Biology, University of Georgia, Athens, GA, USA’ but should have been ‘Martu Elder, Kulyakartu Aboriginal Corporation, 76 Wittenoom St, East Perth, Western Australia 6004’. Affiliation 6 for Tanisha M. Williams was incorrectly given as ‘Department of Biology, Bucknell University, Lewisburg, PA, USA’ but should have been ‘Department of Plant Biology, University of Georgia, 2502 Miller Plant Sciences, Athens, GA 30602-7271’. The affiliation details for Peter M. Veth were incorrectly given as ‘School of Social Sciences, The University of Western Australia, Crawley, WA, Australia’ and ‘ARC Centre of Excellence for Indigenous and Environmental Histories and Futures, University of Western Australia, Crawley, WA, Australia’ but should have been ‘The University of Western Australia, School of Social Sciences and ARC Centre of Excellence for Indigenous and Environmental Histories and Futures, Crawley, WA, Australia’.

The original article has been corrected.

