# Supplementary material for: Seed dispersal by Martu peoples promotes the distribution of native plants in arid Australia
Source: Nat Commun. 2024 Jul 17;15:6019. doi: 10.1038/s41467-024-50300-5 (PMC11255322; doi:10.1038/s41467-024-50300-5)
Supplement: Supplementary file 1 — Description of Additional Supplementary Files [file 41467_2024_50300_MOESM1_ESM.pdf]

Description of Additional Supplementary Files

File Name: Supplementary Data 1
Description: Table with details of fit parameters used in the model selection phase for the top ten best models for presence (hierarchical binomial logit GLM) and abundance (hierarchical poisson GLM) for each species (model rank, AIC values, Akaike weights) along with a list of the model covariates.
