# Supplementary material for: Seed dispersal by Martu peoples promotes the distribution of native plants in arid Australia
Source: Nat Commun. 2024 Jul 17;15:6019. doi: 10.1038/s41467-024-50300-5 (PMC11255322; doi:10.1038/s41467-024-50300-5)
Supplement: Supplementary file 4 — Reporting Summary [file 41467_2024_50300_MOESM4_ESM.pdf]

nature portfolio

Corresponding author(s): Rebecca Bliege Bird

Last updated by author(s): 2024-5-28

Reporting Summary

Nature Portfolio wishes to improve the reproducibility of the work that we publish. This form provides structure for consistency and transparency in reporting. For further information on Nature Portfolio policies, see our Editorial Policies and the Editorial Policy Checklist.

Statistics

For all statistical analyses, confirm that the following items are present in the figure legend, table legend, main text, or Methods section.

n/a	Confirmed	
☐	☒	The exact sample size (*n*) for each experimental group/condition, given as a discrete number and unit of measurement
☐	☒	A statement on whether measurements were taken from distinct samples or whether the same sample was measured repeatedly
☐	☒	The statistical test(s) used AND whether they are one- or two-sided<br>*Only common tests should be described solely by name; describe more complex techniques in the Methods section.*
☐	☒	A description of all covariates tested
☒	☐	A description of any assumptions or corrections, such as tests of normality and adjustment for multiple comparisons
☐	☒	A full description of the statistical parameters including central tendency (e.g. means) or other basic estimates (e.g. regression coefficient) AND variation (e.g. standard deviation) or associated estimates of uncertainty (e.g. confidence intervals)
☐	☒	For null hypothesis testing, the test statistic (e.g. *F*, *t*, *r*) with confidence intervals, effect sizes, degrees of freedom and *P* value noted<br>*Give P values as exact values whenever suitable.*
☒	☐	For Bayesian analysis, information on the choice of priors and Markov chain Monte Carlo settings
☐	☒	For hierarchical and complex designs, identification of the appropriate level for tests and full reporting of outcomes
☐	☒	Estimates of effect sizes (e.g. Cohen's *d*, Pearson's *r*), indicating how they were calculated

Our web collection on statistics for biologists contains articles on many of the points above.

Software and code

Policy information about availability of computer code

Data collection	No software was used to collect data
Data analysis	ArcGIS Pro v. 2.5, QGIS 3.14, R 4.2.2, MuMIn package in R v 1.47.1

For manuscripts utilizing custom algorithms or software that are central to the research but not yet described in published literature, software must be made available to editors and reviewers. We strongly encourage code deposition in a community repository (e.g. GitHub). See the Nature Portfolio guidelines for submitting code & software for further information.

Data

Policy information about availability of data

All manuscripts must include a data availability statement. This statement should provide the following information, where applicable:

- Accession codes, unique identifiers, or web links for publicly available datasets
- A description of any restrictions on data availability
- For clinical datasets or third party data, please ensure that the statement adheres to our policy

Source data obtained on Martu Native Title (plant abundance surveys, archaeological site and water source locations, foraging focal follow observations, fire history mapping) are available under restricted access due to the need for each user to sign intellectual property agreements with the Jamukurnu Yapalikurnu Aboriginal Corporation. Access may be obtained by request of the corresponding author (Rebecca Bliege Bird, rub33@psu.edu) after consultation with JYAC. The remainder of the data used in this analysis is derived from publicly available datasets including the Soil and Landscape Grid of Australia and Landsat-derived vegetation fractional

cover datasets from the TERN repository (https://portal.tern.org.au); the SRTM digital elevation model from the US. Geological Survey (https://earthexplorer.usgs.gov), and archived rainfall data available through the Australian Bureau of Meterology (https://bom.gov.au). Occurrence records (not including Martu-owned data) for all four species are available through Atlas of Living Australia (https://www.ala.org.au).

Research involving human participants, their data, or biological material

Policy information about studies with human participants or human data. See also policy information about sex, gender (identity/presentation), and sexual orientation and race, ethnicity and racism.

Reporting on sex and gender	This information was not collected for this particular study.
Reporting on race, ethnicity, or other socially relevant groupings	The relevant ethnicity of the particpants is described, but it is not relevant to the study.
Population characteristics	As above.
Recruitment	Participants were self selected.
Ethics oversight	This is a long term ethnographic project with multiple funding sources, so oversight was provided by multiple institutions, including the University of Maine, Stanford University, and Pennsylvania State University

Note that full information on the approval of the study protocol must also be provided in the manuscript.

Field-specific reporting

Please select the one below that is the best fit for your research. If you are not sure, read the appropriate sections before making your selection.

☐ Life sciences ☐ Behavioural & social sciences ☒ Ecological, evolutionary & environmental sciences

For a reference copy of the document with all sections, see nature.com/documents/nr-reporting-summary-flat.pdf

Ecological, evolutionary & environmental sciences study design

All studies must disclose on these points even when the disclosure is negative.

Study description

We explore the anthropogenic and environmental factors influencing species distributions with an information theoretic model selection approach in R (v 4.2.2) predicting plant presence/absence using generalized linear mixed models (glmer) for the binomial distribution in the lme4 (v 1.1) package. Because we had many, often collinear, non-anthropogenic climatic and geophysical covariates that could obscure the relationship with our anthropogenic predictors, our variable selection approach followed best practices outlined in Tredennick et al.. We first screened the potential set of non-anthropogenic covariates for associations with the presence of each species, and then checked for any significant collinearities. This initial process identified a total of 9 of the most informative, non-collinear soil/climate and fire-related covariates: soil carbon, percentage green ground cover, percent sand, long term average NDMI, elevation, percent total green+brown vegetation, time-since-fiire, fire frequency since 1973, and season of most recent fire (winter vs summer). These were retained, along with our anthropogenic predictors, in the model selection approach. All binomial presence models included a random effect (transect, n=10) to control for spatial covariance in the transect plots; poisson models for abundance included individual level random effects as recommended to reduce overdispersion. After running each model and checking model diagnostics for spatial autocorrelation and fit, we used the MuMIn package (v 1.47.1) in R to calculate AIC, BIC, model weights and Nakagawa's pseudo R2 values.

The model selection procedure to identify the best models for both presence and abundance involved four steps. First, we ran models predicting species presence with only the random effect, then with each single covariate + random effect. Secondly, we combined all variables of each type (anthropogenic, fire, and soil) into three separate global models (considering only first order interactions), removing the poorest performing covariate until we reached a minimum AIC value. Third, we explored combinations of anthropogenic and fire variables, fire and soil variables, and anthropogenic and soil variables. Fourth, we combined all covariates and predictors into a single model, again removing the poorest performing until we reached a minimum AIC value. Details of all models and model AIC ranks and weights can be found in Supplementary Data 1.

Research sample

1. Presence and abundance records derived from transect surveys of four co-occurring early-mid successional edible plants common to the Western Desert of Australia: Solanum diversiflorum, Solanum centrale, Eragrostis spp., Scaevola parvifolia. 2) Ethnographic observations of plant collection and seed dispersal. 3) Surveys of plant consumption sites to test whether Solanum diversiflorum was growing after seed was seen to be dispersed there.

Model covariates used to predict plant presence and abundance were derived from existing datasets as follows:

Measures of past landscape use: Distance to ethnohistoric site, water permanence, and site type
To operationalize questions about how past legacies of land use may shape landscape-level distribution of key plants, we ask whether the locations of contemporary plant patches are affected by distance to both ethnohistoric and archaeological sites. Ethnohistoric sites are sites that are known in living memory as old residential sites; archaeological sites are those that have a known archaeological component whether or not people remember using them as residential sites in the past. We do not distinguish in our

analysis between the two types of sites. Before their homeland exodus, Martu utilized hundreds of residential campsites and foraging camps within the study area. While visits to more remote sites declined during the mid-20th century hiatus, Martu resumed frequenting many of these sites upon returning to their homelands. Most of the sites in the study area are within daily travel range of the remote communities and are still utilized for both short-term foraging camps and longer multi-day camping trips. While the earliest use of most of these sites (ethnohistoric and archaeological) is not known, the depth of Martu ancestral ties in this region is profound, with occupation of these landscapes extending well into the Late Pleistocene as far back as 48k years ago. The sample of sites in this analysis were all in use prior to Martu exodus from their homelands in the 1960s, meaning that all have had at least some use within the last 70 years, and many still continue to be used in the course of regular foraging activities.

To evaluate the legacy effect of anthropogenic land use on contemporary plant distribution, archaeological site locations from publicly available datasets (n=192) were supplemented with informant recall of residential and foraging camp locations (n=179 sites), and water sources known on topographic maps likely to be used as camping places (n=21 sites). To capture any missing and potentially significant residential sites, we also surveyed high resolution satellite imagery within a 20 km radius of the survey location for any significant water sources not already recorded, mainly soaks, springs, pools and larger rockholes. Using expert knowledge, including our own on the ground observations, each water source was associated with a relative permanence value, 1: ephemeral, to 4: nearly year-round availability. Camping sites and potable water sources are highly correlated: out of 192 sites with known archaeology, the median distance to the nearest water source was 296 meters, and 90% of all sites in our database are within 3 km of water. We are confident in assuming that water sources for which little information is recorded are likely to be potential camping places. To cross-check the campsite surveys, we used an aerial photo mosaic from 1953 which provided coverage for 366 campsite locations. 86% of sites showed signs of use in the form of nearby anthropogenic fire mosaics. We employ those historic photo mosaics and interviews with elders to assist in classifying each site according to its type of use. Major sites (n=74) were defined as such based on ethnographic knowledge of the location as a major meeting place, ceremonial site, or aggregation site and/or visible signs of extensive fire mosaics in 1953. Where detailed archaeological mapping, sampling and dating (both absolute and relative) had occurred66 this was cross-checked with information from Martu elders. The remaining sites were assigned as minor sites (n=376). Informant knowledge of such sites is likely to be biased to those locations frequented by the few people to remain in the desert post 1950. The only potential sites missing from this analysis would be those which are associated with water sources invisible on satellite imagery that were abandoned prior to the 1950's and for which there is no ethnographic or archaeological knowledge. However, given that the plant sampling transects were solely within regions well known to informants and to many of the authors, it is unlikely that this bias affects our results. To obtain the distance to nearest ethnohistoric site from each survey location, we performed a nearest neighbor analysis in ArcGIS Pro v. 2.597) on the center points layer of each 30 meter transect interval and the center points of the ethnohistoric site layer.

Measure of present landscape use: winter fire density
We measured hunting intensity by summing the density of remotely sensed winter season fire footprints as visible in Landsat imagery over a 30-year period prior to the survey date (1973-2003). We reconstructed the fire history of the study region using a time series of 75 30-m resolution Landsat 2-7 image mosaics taken at roughly 6 month intervals (October and April) between 1980 and 2003. Two additional time steps were available from 1973 and 1979, which were used to attach an approximate age to the fires visible in the 1980 base image. Between 1987 and 2003, surface reflectance normalized burn ratio imagery was available from USGS-ESPA, from which we constructed difference images to highlight only those fires burning in each time step. Between 1973 and 1987, fires were detected using the difference of band 4 between time steps. Fires were hand-drawn using the pixel-based region-growing algorithm in QGIS 3.1498 and each time step was converted to a raster image. For our landscape use proxy, only winter fires were counted as there are no non-anthropogenic sources of fire during that season. Winter fire footprints are small and easily represented by a single center-point; we converted the point layer to a raster fire density layer using a kernel density estimation with a 3 km radius. We then extracted fire density for each presence point by using a point sampling function to query the raster layer.
Fire covariates: time-since-fire, time-since-fire diversity, fire frequency, proportion of ground cover

Fire regimes were assessed using the Shannon diversity index of time-since-fire (TSF) age classes, which are calculating using remotely sensed 20-year fire history maps74. To construct the fire history maps in QGIS 3.14, we stacked each raster fire footprint between 1983 and 2003 with the most recent fires on the top layer. Each layer was given a value corresponding to the elapsed months since time zero in 6-month intervals (with Oct-April 1983 being time zero), such that the resulting image maps out the time since last fire of each landscape patch. Martu landscape use has a direct impact on Shannon diversity measures, increasing diversity close to communities where people forage and burn more actively74. Shannon diversity was measured at the 3km scale in a circular region centered on each 30 meter plot centroid as this scale best differentiates anthropogenic fire regimes.

All four plant species also exhibit declining density with time-since-fire (see Table 2). TSF for each transect plot was assessed in two ways: on the ground, by experienced researchers estimating months since last fire, and secondly, by utilizing this ground classification in conjunction with fire history maps to convert the categorical classification and estimations into months since last fire. As the resolution of the satellite imagery used to derive the fire maps was a maximum of 30 m, and the classification maps were at best only 90% accurate, there were some discrepancies between ground and satellite-based estimation of TSF, especially at the fire boundary; these were resolved by using the ground estimation to adjust the boundary of each TSF age class.

Plant presence may also be affected by the frequency of fire, which is influenced by the interval between fires. Martu burning increases the diversity of fire frequencies, creating patches close to the community where fire is both more frequent and less frequent75. Fire frequency at the time of survey was estimated using the fire history maps, as the number of times burnt in the 30 years prior to the survey.

Time since fire is only a rough proxy of successional vegetation structure, as rainfall controls the rate at which plants grow after fire99,100. To account for this, we also added a covariate describing the relative proportion of green, brown and total vegetation sampled at each plot centroid. Green vegetation increases with rainfall and is dominant soon after fire, while brown vegetation consists primarily of senescent Triodia grass hummocks and dominates five to ten years after fire. These raster layers were created in QGIS 3.14 from Digital Earth Australia 30 m Landsat-derived fractional cover datasets available through the Terrestrial Ecosystem Research Network.

Soil covariates: Soil moisture, rainfall, soil components, slope, and aspect
Soil water limitations combined with microgeological variation in nutrient availability are two fundamental drivers in the distribution of endemic plant species across Australia. To help control for this variation in water and nutrient availability, we included several

topographic elements (e.g., slope and aspect) and soil attributes (e.g., water-holding capacity, clay content and sand content) within our analysis. For the terrain features, we derived slope (degrees) and aspect (the compass direction of the slope) values at each sample location in ArcGIS Pro 2.5 from a 1 second SRTM digital elevation model (DEM, resampled to 30 m) provided through the USGS. Both slope and aspect have been shown to influence the distribution of plants in semi-arid regions through the creation of microclimates (e.g., soil temperature, evapotranspiration, wind speed, etc.), the alteration of soil properties (e.g., organic matter context, soil depth, texture, etc.), and the control of hydrological processes (e.g., runoff dynamics, soil water retention, etc.). Soil properties were available as a set of 30 m raster layers available through the Soil and Landscape Grid of Australia. Those that we included in the initial parameter selection were organic carbon, available water capacity at 0-5 cm, percentage clay, silt, phosphorus, and sand.

To capture variability due to persistent soil moisture, we calculated NDMI (the normalized difference water index, a measure of vegetation moisture) as a long-term average measure (since 1987), using 30 m Landsat imagery available as a derived product from the EROS data archive. We also used cumulative rainfall for the 12 months prior to the survey date, drawing on 5 km scale gridded (raster) monthly rainfall datasets available from the Australian Bureau of Meteorology. The grids were resampled in QGIS 3.14 to 30 meters, summed over the relevant 12-month period for each survey date and the total rainfall amount at each survey center point was extracted using the raster point sampling tool.

Sampling strategy

Ten 10-km corridor transects of 10 meter width aggregating stem counts of all four species at 30 meter intervals (n=3130 plots). Transects were chosen with central points near previously visited camping places, radiating outward five km in two directions. Transect location was also stratified to cover a wide range of intensities of Martu landscape use. Transect length was a compromise between time/safety and the number of detections of the target species and was as long as possible given field conditions.

Data collection

The ethnographic and ecological work reported in this study began in June of 2000 by DWB and RBB as part of a long-term project centered on understanding the socio-ecological dynamics of human environment interactions. As part of this project, we quantitatively recorded observations of time allocation, production, distribution, and cultural burning associated with a broad set of foraging activities and land use by Martu men and women. The foraging data used in this study were obtained by accompanying a foraging party leaving the community over a cumulative 800-day observation period between June 2000 and August 2017. Martu typically use vehicles to access the day's foraging region. On arrival they establish a temporary foraging camp (referred to as mirrka ngurra) from which people usually depart on foot, solo or in small cooperative groups. Depending on season and habitat, women most often engage in burning patches of older growth spinifex grass to facilitate their monitor lizard hunting, while men focus much of their efforts in searching for bustards or hill kangaroo. After the day's foraging, people gather back at the hearth of the foraging camp to process, cook, share, and consume the day's catch before returning to the community in the evening. During foraging trips, we recorded all foraging time allocation, yields, and distribution of bush foods among all participants, along with focal follows of individual foragers recording all search, pursuit, capture, transport, and processing. These data comprise a total of 385 trip-days averaging 8 people per trip and 160 unique individuals (80 F, 80 M). We recorded Solanum harvesting time allocation, yields, and sharing on a total of 28 of these trips (Fig 1E), although we participated in many more Solanum harvests than were recorded quantitatively.

Occurrence records for all four species place the study area well within each species' known range (Fig 1), with the caveat that our study area is very remote, roadless, and poorly sampled. Our own surveys show all four species to be common and widely distributed in spinifex sandplain habitat across the entire study area. Plant presence was assessed using pedestrian surveys of plant distributions conducted in June and July of 2003. These surveys employed ten 10 km x 10-meter belt transects to assess presence, absence and abundance via stem counts (see Table 2). Transects radiated outward in two directions from locations centered at ten past and present camping places so that we could more accurately capture the effect of distance from site on plant presence and abundance. Transect location was also stratified according to landscape use intensity, with two transects near ethnohistoric sites in little used landscapes, two near sites in heavily used landscapes, and the remainder near sites in landscapes with moderate use. Stem counts (counting main stem only) were aggregated to 300 m2 in the analysis to more closely match the 30 m pixel resolution of the remotely sensed imagery, producing .

In 2018, we selected 12 foraging camps (hearth locations used to consume bush foods between 2002 and 2017) for which we had complete quantitative foraging records to survey for the presence and abundance of S.diversiflorum (Fig 1F). Half of these sites (controls, n=6) had no S.diversiflorum consumption recorded during use. At the 6 remaining sites we had detailed records of S.diversiflorum harvesting, consumption, and seed dispersal, and the hearth was located in an area where no plants were growing at the time of use. Each foraging camp dispersal survey consisted of one 50x50 m plot centered at the camp hearth, and one randomly placed comparison plot located at least 500 meters from the hearth.

All data was recorded using pen/pencil and paper in notebooks or on paper data recording sheets.

Timing and spatial scale

Transect surveys were conducted in June and July of 2003. Rainfall was higher than normal in the prior three years which maximized our probability of finding plants. The spatial scale of transect surveys was extensive, with the farthest transects more than 100 km apart (see Figure 1 F).

The dispersal site surveys were conducted in July 2018 also during the austral winter. Rainfall was below average for this survey, and it may have impacted our ability to find plants, but all sites were affected equally by the lack of rain. The spatial scale of these surveys was limited to the selected sites where Solanum diversiflorum was known to be consumed (or not), all of which were close to Parnngurr community. A 50 meter plot centered on the hearth was used to assess plant presence/abundance, which was of sufficient size to capture most of the discard and fruit processing behavior without adding additional uncertainty or habitat diversity. The corresponding author's fieldnotes and photographs of the camp were used to confirm that no plants were present before that temporary hearth was occupied and used to clean fruit.

The foraging observations were conducted over a period of ten years between 2000 and 2017 during both Austral winter and summer, covering periods of very high and very low rainfall. Precise estimates of rainfall are not possible as there are no climate recording stations (the nearest is 140 km distant). Foraging observations covered the spatial range of Martu Native Title, at locations chosen by participants.

Data exclusions	The analyzed sample includes only plots falling in majority habitat burnt less than 3.5 years prior (n=2429) because nearly all presences were within this habitat type.
Reproducibility	Not applicable as this data is based on field observations.
Randomization	Not relevant as this data is based on field observations.
Blinding	Not relevant as this data is based on field observations.

Did the study involve field work? ☒ Yes ☐ No

Field work, collection and transport

Field conditions	The transect data was collected in June and July of 2003 over a period of six weeks during the Austral winter. Temperature and rainfall were typical of the winter months in the region. The dispersal site surveys were conducted in 2018 also during the austral winter. The foraging observations were conducted over a period of 17 years during both Austral winter and summer.
Location	Fieldwork was conducted on Martu Native title, primarily around Parnngur community -22.817072437076867, 122.5976767109026
Access & import/export	Only occurrence records were recorded. No samples were imported or exported. Access to the field data collection was granted by permission from the Martu Prescribed Body Corporate (now JYAC). No other permissions are necessary for ecological survey without sample collection.
Disturbance	There was no disturbance. Transects were conducted on foot.

Reporting for specific materials, systems and methods

We require information from authors about some types of materials, experimental systems and methods used in many studies. Here, indicate whether each material, system or method listed is relevant to your study. If you are not sure if a list item applies to your research, read the appropriate section before selecting a response.

Materials & experimental systems

n/a	Involved in the study	
☒	☐	Antibodies
☒	☐	Eukaryotic cell lines
☐	☒	Palaeontology and archaeology
☒	☐	Animals and other organisms
☒	☐	Clinical data
☒	☐	Dual use research of concern
☐	☒	Plants

Methods

n/a	Involved in the study	
☒	☐	ChIP-seq
☒	☐	Flow cytometry
☒	☐	MRI-based neuroimaging

Palaeontology and Archaeology

Specimen provenance	no specimens were collected for the purposes of this study
Specimen deposition	see above
Dating methods	see above

☐ Tick this box to confirm that the raw and calibrated dates are available in the paper or in Supplementary Information.

Ethics oversight	the archaeological data used in this study is derived from already existing sources.

Note that full information on the approval of the study protocol must also be provided in the manuscript.

Dual use research of concern

Policy information about dual use research of concern

Hazards

Could the accidental, deliberate or reckless misuse of agents or technologies generated in the work, or the application of information presented in the manuscript, pose a threat to:

No	Yes	
☒	☐	Public health
☒	☐	National security
☒	☐	Crops and/or livestock
☒	☐	Ecosystems
☒	☐	Any other significant area

Experiments of concern

Does the work involve any of these experiments of concern:

No	Yes	
☒	☐	Demonstrate how to render a vaccine ineffective
☒	☐	Confer resistance to therapeutically useful antibiotics or antiviral agents
☒	☐	Enhance the virulence of a pathogen or render a nonpathogen virulent
☒	☐	Increase transmissibility of a pathogen
☒	☐	Alter the host range of a pathogen
☒	☐	Enable evasion of diagnostic/detection modalities
☒	☐	Enable the weaponization of a biological agent or toxin
☒	☐	Any other potentially harmful combination of experiments and agents

Plants

Seed stocks	No seed was collected.
Novel plant genotypes	Not applicable
Authentication	Not applicable.
